# A confocal microscopic study on biofilm formed by Pseudomonas spp. isolated from lower respiratory tract infection from HIV and non-HIV populations

**DOI:** 10.1186/1471-2334-12-S1-P56

**Published:** 2012-05-04

**Authors:** C Anitha, A Santhoshkumar, Padma Krishnan, BVR Tata, S Senthamarai, S Sivasankari, V Venugopal, Rajasekharan Sikhamani, M Pushkala, SK Amshavathani

**Affiliations:** 1 Dept of Microbiology (Faculty of Medicine), Dr. ALM PGIBMS, University of Madras, Taramani, Chennai-113, India; 2 LIFE/P.G and Research Department of Advanced Zoology and Biotechnology, Loyola College, Chennai, India; 3 Condensed Matter, Physics Division, Indira Gandhi Centre for Atomic Research, Kalpakkam-603 102, India; 4 Meenakshi Medical College and Research Institute, Kanchipuram, Tamil Nadu, India; 5 Government Hospital of Thoracic Medicine, Tambaram Sanatorium, Tambaram, Chennai, India; 6 Department of Immunohaemotology, Dr. M.G.R Medical University, Guindy, Chennai, India

## Background

*Pseudomonas* is one of the predominant microorganisms of chronic lung infections. Bacteria growing in biofilm often develop multicellular, three-dimensional structures known as micro colonies. *Pseudomonas* colonizes the lungs by forming biofilm micro colonies throughout the lung. In this study, using *Pseudomonas spp*. artificial biofilm was grown on flow cell chamber and images were taken using Confocal Laser Scanning Microscope (CLSM), to establish an experimental model for artificial biofilm in vitro.

## Methods

Out of 71 isolates analysed, 45 were HIV and 24 non-HIV isolates. 19 (26.76%) produced biofilm. 6/45 (13.33%) HIV isolates and 13/24 (54.17%) non-HIV isolates were biofilm producers. 4 strains were taken to study the in vitro biofilm formation using flow cell chamber and their 3D structure and architects were studied using CLSM. Biofilm formation was monitored at different time intervals (3, 72 and 144 h). For each time interval, one channel was stained with acridine orange dye and the images obtained were quantitatively analyzed by COMSTAT.

## Results

A time lapse study of various time intervals (3, 72 and 144 hrs) were taken to study the biofilm formed by the *Pseudomonas aeruginosa*. We therefore used a computer program COMSTAT for quick and easy analysis of the biofilm image data which calculates a number of variables characterizing the three-dimensional structures.

## Conclusion

Biofilm have been established as a main cause of infections due to the increased chemo resistance compared with bacteria in suspensions**.** Hence it is necessary to characterize the developmental steps leading to the formation of the *P. aeruginosa* biofilm.

